# Mucoadhesive Nanostructured Lipid Carriers of Ketoconazole for Enhanced Dermal Delivery and Antifungal Activity: Formulation Optimization and In Vivo Evaluation

**DOI:** 10.3390/pharmaceutics18060753

**Published:** 2026-06-19

**Authors:** Mashan Almutairi, Ahmed Adel Ali Youssef, Yazed S. Alsowaida, Ahmed Alobaida, Samir A. Ross

**Affiliations:** 1 Department of Pharmaceutics, College of Pharmacy, University of Ha’il, Ha’il 81442, Saudi Arabia; a.alobaida@uoh.edu.sa; 2 Department of Pharmaceutics & Pharmaceutical Technology, Faculty of Pharmacy, Kafrelsheikh University, Kafrelsheikh 33516, Egypt; eldiasti89@pharm.kfs.edu.eg; 3 Department of Clinical Pharmacy, College of Pharmacy, University of Ha’il, Ha’il 81442, Saudi Arabia; ysowaida@gmail.com; 4 National Center for Natural Products Research, School of Pharmacy, The University of Mississippi, University, MS 38677, USA; sross@olemiss.edu

**Keywords:** ketoconazole, nanolipid carriers, fungal, in vivo, flux, ex vivo, irritation, permeation

## Abstract

**Background/Objective:** Topical therapy remains a cornerstone in managing fungal infections due to the deep-seated nature of the pathogens and the persistence of the disease. Ketoconazole (KTZ) is a broad-spectrum antifungal agent, but its highly lipophilic nature presents considerable challenges in developing effective topical formulations. Additionally, oral KTZ has been subject to labeling restrictions and market withdrawal due to its association with severe hepatic adverse effects. This study was conducted to design, optimize, and evaluate KTZ-loaded nanolipid carriers (NLCs; KTZ-NLCs) as a delivery platform that could improve cutaneous bioavailability and enhance antifungal activity. **Methods:** The optimized KTZ-NLCs were further incorporated into a mucoadhesive system (KTZ-NLCs-C) through the inclusion of Carbopol^®^ 940 NF, aiming to improve the retention of the formulation on the skin surface. NLCs were characterized in terms of their physical appearance, particle size, polydispersity index, zeta potential, pH, viscosity, drug content, and entrapment efficiency. The optimized KTZ-NLC and KTZ-NLCs-C formulations were subsequently assessed for in vitro drug release, ex vivo skin permeation and deposition, as well as in vivo skin irritation. **Results:** In vitro release studies revealed that nanocarrier systems provided a sustained release of KTZ over 24 h. The ex vivo transdermal flux and permeability coefficient of KTZ from the lead KTZ-NLCs-C formulation were approximately 2.8-fold greater than those achieved with the marketed cream formulation. The in vivo skin irritation studies indicate that NLC-based formulations are suitable for topical applications. The lead formulation was stable for 90 days (the final time point evaluated) under refrigerated and room-temperature storage conditions. **Conclusions:** These findings suggest that the NLC-based system is a promising platform for the topical delivery of KTZ and has the potential to enhance the therapeutic outcomes for patients with superficial fungal infections.

## 1. Introduction

The rising prevalence of superficial fungal infections, coupled with growing antifungal resistance, poses a significant global health concern and imposes a substantial economic impact. Fungal infections involving the skin and its adnexal structures, including hair and nails, are prevalent worldwide. Over the past four decades, substantial progress has been achieved in their management. Historically, therapeutic options were largely confined to antiseptic agents with limited antifungal efficacy; however, modern clinical practice is now supported by a broad and continually evolving arsenal of targeted antifungal therapies [1]. Although the evolution of these therapies has not been without challenges, issues such as antimicrobial resistance—commonly observed with antibacterial agents—have exerted relatively limited influence on current antifungal regimens. A notable exception is superficial *Candida* infections, where azole resistance is well documented [1]. This phenomenon, coupled with selection pressure, has facilitated the emergence of species such as *Candida glabrata*, characterized by reduced azole susceptibility. Furthermore, the emergence of *Candida auris* as a multidrug-resistant pathogen represents an additional concern, although its impact on cutaneous infections remains minimal despite frequent reports of superficial colonization [1]. Drug-related toxicity is generally uncommon; however, when present, even infrequently, it poses a significant risk-benefit dilemma in the context of treating non-life-threatening conditions. This contrasts with systemic mycoses, as exemplified by the regulatory withdrawal of oral ketoconazole (KTZ) for superficial infections in both Europe and the United States [1].

The introduction of KTZ (Nizoral^®^) in 1977 by Janssen Pharmaceutica marked a significant milestone in medical mycology as the first broad-spectrum oral antifungal agent. In July 1981, KTZ received approval from the FDA for the treatment of systemic fungal infections. For nearly a decade thereafter, it remained the sole oral antifungal option available for managing systemic mycoses [2]. Oral KTZ has been subjected to labeling changes and, in certain cases, withdrawn from the market owing to its association with serious hepatic adverse effects [2]. However, topical KTZ remains widely regarded as both effective and safe for managing superficial fungal infections. Furthermore, emerging dermatologic applications for topical KTZ have been identified, including its use in conditions such as onychomycosis, blepharitis, and certain forms of alopecia [3].

KTZ is a broad-spectrum imidazole antifungal agent that exerts its activity by disrupting ergosterol biosynthesis through inhibition of a cytochrome P450-dependent enzyme, thereby altering the structural integrity and functional properties of the fungal cell membrane, consistent with the mechanism of other imidazole derivatives. Early in vitro investigations demonstrated KTZ’s efficacy against a wide range of organisms, including dermatophytes, yeasts, molds, and dimorphic fungi, as well as certain bacterial species. Additionally, in vivo studies confirmed its therapeutic potential in animal models of oral, vaginal, cutaneous, and systemic candidiasis [4,5,6,7].

According to the Biopharmaceutical Classification System (BCS), KTZ falls under Class II, characterized by low aqueous solubility but high permeability [8]. KTZ exhibits high lipophilicity (log P ≈ 4.74) and very limited aqueous solubility (~0.04 mg/mL), which pose considerable challenges in formulating effective topical systems capable of achieving sufficient drug delivery across the stratum corneum barrier [9]. KTZ is pharmaceutically marketed as shampoo and cream drug products. Conventional cream formulations are often associated with limited drug penetration to the target site, adverse effects such as swelling, irritation, erythema, pruritus, and contact dermatitis, and inadequate therapeutic efficacy for managing deeper skin infections [9,10]. Likewise, the primary disadvantages of applying drugs in a shampoo formulation are the limited contact time for the drug to be absorbed and an increased risk of localized side effects like skin irritation and dryness. Therefore, enhancing permeation at the site of fungal infection, together with achieving a controlled and sustained release profile, is highly desirable for the effective topical delivery of KTZ.

Topical drug delivery is an important route used for the administration of pharmaceutical products. However, limited drug penetration due to the barrier nature of the stratum corneum layer is a major problem associated with this route and necessitates searching for biocompatible drug carriers that will enable deeper drug penetration into skin layers for improved therapeutic outcomes. Nanolipid carriers (NLCs) consist of a matrix of solid and liquid lipids dispersed in an aqueous solution of surfactants [11]. These nanocarriers have several advantages, such as biocompatibility, high entrapment efficiency, organic solvent-free, and low cytotoxicity [11]. Moreover, NLCs have been shown to facilitate enhanced drug permeation across biological membranes, improve formulation stability, and have been successfully utilized in many oral, pulmonary, intravenous, ocular, and dermal drug delivery applications [11,12].

NLCs represent a promising strategy for the topical delivery of poorly water-soluble drugs such as KTZ by encapsulating the drug within a lipid-based matrix. In the current study, KTZ-NLCs were developed and optimized to enhance KTZ solubility and improve skin permeability. Production parameters such as particle size and zeta potential were optimized using a full factorial design experiment. The optimized KTZ-NLCs were transformed into KTZ-NLCs-C by incorporating a gelling agent. Further, optimized KTZ-NLCs and KTZ-NLCs-C formulations were evaluated for stability, ex vivo skin permeation and deposition, and in vivo skin irritation studies against the marketed cream and an in-house-prepared suspension formulation as the control in an animal model.

## 2. Materials and Methods

### 2.1. Materials

KTZ was acquired from TCI^®^ chemicals (Tokyo Chemical Industry, Portland, OR, USA). Polyethylene glycol 200 (PEG 200) was purchased from Sigma Aldrich (St. Louis, MO, USA). Precirol^®^ ATO 5, Gelucire^TM^ 43/01, Gelucire^TM^ 44/14, Gelucire^TM^ 50/13, and Compritol^®^ 888 ATO were generous gifts from Gattefossé Corporation (Paramus, NJ, USA). Carbopol^®^ 940 NF grade was acquired from Spectrum Chemicals (New Brunswick, NJ, USA). Labrafac PG (Propylene glycol dicaprolate) was obtained from Gattefossé India Pvt. Ltd. (Saint Priest, France). PEG 400 was a kind gift from BASF Corporation (Florham Park, NJ, USA). Tween^®^ 80, Oleic acid, and Ultra-Fast liquid chromatography (UFLC) grade solvents such as methanol and acetonitrile were obtained from Fischer Scientific (Hampton, NH, USA). All screened oils were purchased from Gattefossé India (Mumbai, Maharashtra, 400070, India). Glassware such as scintillation vials, centrifuge tubes, and UFLC vials was acquired from Fischer Scientific (Hampton, NH, USA).

### 2.2. Methods

#### 2.2.1. Analytical Method

Samples were analyzed for KTZ using a previously reported RP-HPLC method with slight modifications [13]. Quantification of KTZ was performed on a Shimadzu UFLC system equipped with a SIL-20AC autosampler, an SPD-M20A UV/VIS photodiode array detector, and an LC-20AD solvent delivery module (Shimadzu Corporation, Nakagyo-Ku, Kyoto, Japan). Chromatographic separation was achieved using reversed-phase chromatography on a Waters Symmetry^®^ C18 column (5 μm, 150 × 4.6 mm, Waters, Milford, CA, USA). The mobile phase consisted of a mixture of acetonitrile and 0.2% triethylamine (45:55 *v*/*v*, pH: 6.4 adjusted with orthophosphoric acid) at a flow rate of 1.0 mL/min. Before analysis, the mobile phase was filtered through a 0.45 µm membrane filter (MF-Millipore™, Saint-Quentin, Yvelines, France) and degassed. The UV detection wavelength was set at 230 nm, and detector sensitivity was set at 1.0 AUFS (Absorbance Units Full Scale). The injection volume was adjusted to 20 μL, and the temperature during this analysis was 25 °C.

#### 2.2.2. Screening of Lipid Excipients

The solubility of KTZ in different oils and solid lipids was evaluated by introducing 5 mg of the drug into 1 g of oil or molten lipid in separate 3 mL glass vials, followed by continuous magnetic stirring at 2000 rpm and 80 ± 2 °C. Each mixture was visually examined to determine whether complete dissolution had occurred. When full solubility was observed, additional KTZ was added in 5 mg portions, with stirring and observation repeated after each step. This iterative process continued until the maximum amount of KTZ that could be dissolved in 1 g of lipid was reached.

#### 2.2.3. Physical Compatibility Between Solid and Liquid Lipid

Solid and liquid lipids selected based on the preliminary screening study were mixed in different ratios (3:1, 3:2, 4:1, and 4:2) in glass vials. The lipid mixture was melted, vortexed, and allowed to congeal at room temperature. The glass vial was visually observed to confirm the absence of layer separation in the congealed matrix.

##### Saturation Solubility in Liquid Lipids

To determine the solubility of KTZ in the candidate liquid lipids, an excess quantity of the drug (300 mg) was added to 1 g of each candidate oil in glass vials and placed in a reciprocating water bath (Precision™, Waltham, MA, USA). The samples were kept at 25 ± 0.5 °C under continuous agitation at 100 rpm for 48 h to reach equilibrium. Following this, the mixtures were centrifuged (13,000 rpm for 20 min) using a Fisherbrand™ AccuSpin 17R centrifuge (Waltham, MA, USA). The supernatant was then carefully collected, passed through a 0.22 µm nylon membrane filter, and subsequently quantified for KTZ content by UFLC after appropriate dilution [14].

##### Saturation Solubility in Solid Lipids

An excess amount of KTZ (300 mg) was added to 1 g of the candidate solid lipid in glass vials. The vials were kept inside a reciprocating water bath that was maintained at 80 ± 0.5 °C and agitated at 100 rpm for 48 h to ensure equilibrium. After incubation, samples (10 µL) were withdrawn from the upper layer of the KTZ–lipid mixture and diluted with 990 µL of methanol. The samples were centrifuged (13,000 rpm for 20 min), and the resulting supernatant was filtered through a 0.22 µm nylon membrane filter. KTZ concentration was analyzed by the UFLC method described above, following appropriate dilution [14].

### 2.3. Experimental Design

The key independent variables (factors) that influence formulation quality and may affect the in vivo biological performance of nanocarrier systems were identified and selected [15]. A full factorial (2^4^) experimental design was utilized to develop the NLC formulations, in which four independent factors were optimized with respect to the responses: mean particle size (PS, nm, R_1_) and zeta potential (ZP, mV, R_2_). A total of sixteen experimental runs were generated using DesignExpert^®^ software (version 8.0.7.1, Minneapolis, MN, USA). The employed factors included solid lipid concentration (A, % *w*/*v*), liquid lipid concentration (B, % *w*/*v*), lipophilic surfactant concentration (Span^®^ 80, C, % *w*/*v*), and hydrophilic surfactant concentration (Tween^®^ 80, D, % *w*/*v*). Each factor was evaluated at two levels: high (+1) and low (−1), corresponding to A: 4.0 and 3.0% *w*/*v*, B: 2.0 and 1.0% *w*/*v*, C: 1.0 and 0.5% *w*/*v*, and D: 3.0 and 2.0% *w*/*v*, respectively. The relatively narrow % *w*/*v* ranges chosen for the solid lipid, liquid lipid, and surfactant concentrations were guided by both formulation feasibility and literature precedence, rather than being arbitrary or restrictive. Preliminary screening studies, solubility assessments, and prior experience with lipid-based nanosystems indicated that only limited concentration windows could produce physically stable, homogeneous NLC dispersions suitable for topical application. Wider excursions beyond these ranges resulted in undesirable outcomes such as lipid phase separation, excessive particle growth, aggregation, or poor zeta potential, which would compromise formulation quality and reproducibility. Moreover, because the objective of the factorial design was optimization rather than broad exploration, the design space was deliberately constrained to a practically relevant region where acceptable NLC characteristics were already expected. This approach is consistent with quality-by-design principles, which recommend defining a focused design space based on prior knowledge to ensure meaningful interpretation of factor effects and robust optimization. The details of the full factorial design experiment are discussed in Table 1 and Table 2.

#### 2.3.1. Preparation of KTZ-NLCs

KTZ-NLCs were prepared by homogenization coupled with the probe sonication method [16]. Briefly, KTZ (1.0% *w*/*v*), along with the solid lipid, liquid lipid, and lipophilic surfactant, was combined during the melting of the solid lipid at 80.0 ± 2.0 °C to form the oil phase. The aqueous phase, consisting of Tween^®^ 80 dispersed in Milli-Q water, was heated to the same temperature as the oil phase. This hot (80.0 ± 2.0 °C) aqueous phase was then slowly added dropwise to the oil phase while maintaining continuous magnetic stirring at 2000 rpm for 10 min to generate a primary emulsion. The resulting emulsion was subsequently subjected to high-speed homogenization (14,000 rpm, 5 min) using a T25 Ultra-Turrax^®^ homogenizer (IKA, Wilmington, NC, USA), producing a hot macroemulsion. This macroemulsion was further processed using a probe sonication instrument (Sonic Dismembrator, Model FB-120, Fisherbrand™) at 40% amplitude with a 3 mm stepped microtip for 10 min, employing a pulsed cycle of 10 s on and 10 s off to obtain the final NLC dispersions.

#### 2.3.2. Preparation of the Mucoadhesive KTZ-NLCs (KTZ-NLCs-C)

The NLC-C formulations were prepared following the same procedure used for NLCs, with a slight modification. The total amount of Milli-Q water was divided equally into two portions. One portion was used to prepare the mucoadhesive polymer (Carbopol^®^ 940 NF) aqueous solution, while the second portion was utilized to prepare the aqueous phase as described previously.

#### 2.3.3. Control Formulations

##### Ketoconazole Cream (KTZ-C)

KTZ cream (2% *w*/*w*), marketed under the brand name Nizoral^®^, is commercially available in Egypt, including in Kafrelsheikh Governorate.

##### Ketoconazole Suspension (KTZ-S)

KTZ suspension (KTZ-S: 10 mL, 1% *w*/*v*) was prepared by dissolving KTZ in Tween^®^ 80 (2.0% *w*/*v*, suspending agent) solution under continuous magnetic stirring (2000 rpm for 1 h) at 40 °C and then 1 h at room temperature [13].

#### 2.3.4. Measurement of Particle Size, Polydispersity Index, and Zeta Potential

The particle size (PS), polydispersity index (PDI), and zeta potential (ZP) of the prepared NLCs were determined using a Zetasizer Nano ZS (Zen3600, Malvern Panalytical Inc., Westborough, MA, USA) at 25 °C in disposable solvent-resistant microcuvettes (ZEN0040, Malvern Panalytical Inc., Westborough, MA, USA) [17]. Prior to measurement, the microcuvettes were carefully rinsed with 0.22 µm filtered Milli-Q water to remove any potential particulate contamination. PS and PDI values were determined based on volume distribution. The samples were diluted at a ratio of 1:100 using filtered Milli-Q water and analyzed in triplicate. The ZP values were obtained using the same diluted samples.

#### 2.3.5. pH Measurement

The pH of all NLC formulations was measured using a Mettler Toledo SevenMulti™ pH meter (Columbus, OH, USA) equipped with a Light Edition sensor probe (LE 438, METTLER TOLEDO^®^, Columbus, OH, USA). Prior to measurement, the pH meter was calibrated using a standard buffer kit with known pH values (Orion™ Standard All-in-One™ buffers at pH 4.01, 7.00, and 10.01; Thermo Fisher Scientific, Chelmsford, MA, USA). This calibration ensured correction of electrode-related variations and improved the accuracy and reproducibility of the readings. All measurements were conducted in triplicate.

#### 2.3.6. Drug Content (Assay)

KTZ content was determined using the lipid precipitation method, wherein the formulation (50 μL) was extracted in methanol (950 μL). The mixtures were centrifuged (AccuSpin 17R centrifuge, Fisher Scientific, USA) at 13,000 rpm for 20 min. Then, the supernatant was collected with a micropipette and analyzed for KTZ content using the analytical method described above. The KTZ content was used to calculate entrapment efficiency.

#### 2.3.7. Entrapment Efficiency (EE)

The EE (%) was determined by quantifying the free, unencapsulated drug present in the aqueous phase of the formulations. Ultrafiltration was performed using 100 kDa centrifugal filter units (Amicon Ultra, Fisher Scientific, Hanover, IL, USA) to separate the free drug from the NLC dispersions. The formulation (500 μL) was added to the sample reservoir and centrifuged (13,000 rpm for 20 min). The filtrate collected in the receiver compartment was analyzed to quantify the KTZ content. All measurements were carried out in triplicate, and the % EE was calculated by using the following equation [18]:

$$\;\mathrm{EE}\;(\%)\;=\left[\frac{\mathrm{KTZ}\;\mathrm{content}-\mathrm{Amount}\;\mathrm{of}\;\mathrm{KTZ}\;\mathrm{in}\;\mathrm{the}\;\mathrm{filtrate}}{\mathrm{Amount}\;\mathrm{of}\;\mathrm{KTZ}\;\mathrm{added}}\right]\times \;100$$

#### 2.3.8. Viscosity Measurement

The viscosity of nanodispersions was measured using a Brookfield cone and plate viscometer (LV-DV-II+Pro Viscometer, Middleboro, MA, USA). Prior to measurement, the gap between the cone and the plate was properly adjusted. A sample volume of 0.5 mL was then placed onto the plate surface. The formulation was maintained at a constant temperature of 25 °C using a circulating water bath. Measurements were conducted using a CPE 52 spindle at a rotational speed of 10 rpm, and viscosity values were recorded in standalone mode. 

#### 2.3.9. In Vitro Release Testing

A mixture of phosphate buffer (pH 7.4) and Tween^®^ 20 (2% *v*/*v*) was employed as the receptor medium for both in vitro release studies and ex vivo skin permeation and deposition experiments [19]. In vitro release of KTZ was performed using a vertical Franz diffusion cell system (Logan Instruments Corp., Somerset, NJ, USA). A cellulose membrane (0.44 mm thickness; 12–14,000 Da pore size) was placed between the donor and receptor compartments, providing an effective diffusion area of 0.636 cm^2^. The receptor compartment was filled with the release medium (5 mL) and maintained under continuous magnetic stirring using a circulating water bath (32.0 ± 0.5 °C). At specified time intervals, 0.5 mL samples were withdrawn from the receptor compartment and replaced immediately with an equal volume of fresh medium to maintain sink conditions. The collected samples were quantified for KTZ using UFLC. Release data were subsequently fitted to different kinetic models (zero-order, first-order, Higuchi, and Korsmeyer–Peppas) using the DDSolver add-in for Microsoft Excel (2019, Microsoft Corporation, Redmond, WA, USA), which is commonly employed for evaluating drug dissolution profiles.

#### 2.3.10. Ex Vivo Permeation

Animals: Male albino rats (*Rattus norvegicus*), weighing between 190 and 250 g, were purchased from the laboratory animal facility of the Egyptian Organization of Biological Products and Vaccines (VACSERA, Helwan, Cairo, Egypt). The animals were housed under standard hygienic conditions, supplied with a balanced diet, and allowed free access to water throughout the study period. Experiment: The rats were provided with unrestricted access to food and water prior to being sacrificed under ether anesthesia. Hair from the abdominal region was removed using a depilatory agent. The skin was then carefully excised, and the underlying subcutaneous tissue was manually separated. The dermal surface was cleaned with isopropyl alcohol to remove any residual fat. The full-thickness skin samples were subsequently rinsed with isotonic phosphate-buffered saline (IPBS), wrapped in aluminum foil, and stored at −20 °C until further use [13].

Both test formulations (KTZ-NLCs and KTZ-NLCs-C) and control formulations (KTZ-S and KTZ-C) were evaluated for ex vivo permeation using a vertical Franz diffusion cell system (Logan Instruments Corp., Somerset, NJ, USA). Prior to the experiment, rat skin samples were allowed to equilibrate to room temperature. The skin was then carefully placed between the donor and receptor compartments, ensuring that the stratum corneum faced the donor chamber containing the formulation. Each formulation was applied to the skin surface within the donor compartment using a syringe. The receptor medium was continuously stirred and maintained at 32.0 ± 0.5 °C using a circulating water bath. At specified intervals over a 6 h period, 0.5 mL samples were withdrawn from the receptor compartment and immediately replaced with an equal volume of fresh medium. The KTZ concentration in the collected samples was subsequently quantified using the previously described UFLC method. The cumulative amount of KTZ permeated (Q_n_), steady-state flux (J_ss_), and transdermal permeability coefficient (P_eff_) were calculated to study the ex vivo permeation of KTZ across the rat skin. The analysis of all samples was conducted in triplicate.

- *Q_n_* was calculated using the following equation:
  $$Q_{n}=V_{r}C_{r(n)}+\sum_{x=1}^{x=n}V_{s(x-1)}C_{r(x-1)}$$
  where n is the sampling time point, V_r_ is the volume of the receiver chamber (mL), V_s_ is the volume of the sample withdrawn from the receiver chamber at the nth time point (mL), and C_r(n)_ is the concentration of KTZ in the receiver chamber at the nth time point. The skin permeation rate (dQ/dt) was determined from the slope of the plot of Q_n_ versus time.
- J_ss_ of KTZ was calculated using the following equation:
  J_ss_ = (dQ/dt)/A
  where Q is the amount of KTZ transported through the skin.
- The transdermal permeability coefficient was calculated using the following equation:
  P_eff_ = J_ss_/C_0_
  where C_0_ is the initial donor concentration for KTZ.

#### 2.3.11. Ex Vivo Deposition Study

Upon completion of the ex vivo permeation experiment, the skin was carefully removed from the diffusion cell. Then, the skin was gently cleansed using cotton swabs moistened with methanol (Q-tips^®^) to remove any residual unabsorbed KTZ from the surface. The effective diffusion area of the skin was then excised, weighed, and cut into small sections before being immersed in methanol. Subsequently, the samples were homogenized in methanol (2 h) to facilitate drug extraction. The resulting homogenate was then centrifuged at 13,000 rpm for 15 min [20]. The supernatant was then collected after centrifugation and injected into UFLC to analyze the KTZ content within the skin tissue homogenate by applying the following equation:

Q_d_ = Q_s_/W_s_

where Q_d_ represents the amount of KTZ deposited per milligram of skin; Q_s_ denotes the quantity of KTZ measured within the effective permeation area; and W_s_ is the weight of that effective permeation area.

#### 2.3.12. Skin Irritation Studies

Skin irritation studies of both lead and control formulations were performed in male albino rats using the Draize patch method [21]. Rats were acclimatized under standard laboratory conditions and housed in polypropylene cages with unrestricted access to a standard diet and water for seven days prior to the experiment. Before initiation of the study, animals were carefully screened for any dermatological abnormalities, and those presenting lesions or acne-like conditions were excluded. The animals were randomly allocated into six groups (N = 4). Hair on the dorsal surface was carefully clipped 24 h before treatment, and the exposed skin was re-examined to ensure normality. The formulations were applied to a defined area (5 ± 1 cm^2^) on the dorsal region as follows: Group I served as the negative control; Group II received optimized KTZ-NLCs (200 μL); Group III was treated with KTZ-NLCs-C (200 μL); Group IV received KTZ-S (200 μL); Group V was administered the marketed cream formulation (100 mg); and Group VI was treated with formalin solution (0.8% *w*/*v*, 200 µL). The application sites were observed over 24 h for any changes in skin color, texture, erythema, and edema, and responses were evaluated relative to the control group using a standardized visual scoring system [13].

#### 2.3.13. In Vitro Antifungal Activity

The antifungal activity of KTZ from the test and control formulations was evaluated against *Candida albicans* cultures, a common opportunistic human fungus, using the cup plate method. The *C. albicans MTCC 227* strain was cultured on 2% Sabouraud agar and maintained on slants at 5 ± 3 °C. For the assay, cultures were subcultured onto fresh slant agar and incubated at 32 °C for 48 h prior to testing. The microbial cells were suspended in 0.9% *w*/*v* normal saline to obtain a suspension with an approximate density of 10^6^ cells/mL. The viable cell count (CFU/mL) was determined using the spread plate technique. A suitable volume of this inoculum (≈10^6^ CFU/mL) was mixed with molten Sabouraud agar to achieve a final concentration of approximately 100 CFU per 30 mL of medium. The prepared medium was then dispensed aseptically into sterile Petri dishes (30 mL per plate) and allowed to solidify. Four wells (1 cm^2^) were created in each plate using a stainless-steel cylinder. These wells were filled with predetermined amounts of the respective formulations—test (KTZ-NLCs, KTZ-NLCs-C), negative control (placebo NLC and NLC-C), and positive control (KTZ-S, KTZ-C)—using a sterile micropipette, ensuring accurate loading without spillage or formation of air bubbles. The plates were incubated at 32 °C for 48 h to allow fungal growth and drug diffusion. Antifungal activity was assessed by measuring the diameter of the inhibition zones surrounding each well. Measurements were recorded in millimeters using a calibrated digital Vernier caliper, and the average of two perpendicular diameters was calculated to represent the antifungal activity.

#### 2.3.14. Physicochemical Stability Studies

A stability study for the lead formulation was initiated under refrigerated (5 ± 3 °C) and room (30 ± 2 °C) temperature storage conditions. The formulation was evaluated for physical instability issues such as cracking and changes in color, PS, PDI, ZP, pH, viscosity, drug content, and EE at predetermined time intervals.

#### 2.3.15. Statistical Analysis

All experiments were performed in triplicate, and results are expressed as mean ± standard deviation (SD). Statistical evaluation was carried out to determine the significance of differences among formulations and experimental conditions. Analysis of variance (ANOVA) was employed to assess the effect of formulation and process variables on the measured responses. When applicable, regression analysis was used to evaluate the relationship between independent and dependent variables. The probability value of *p* < 0.05 was considered statistically significant. Statistical analysis and model generation were performed using SPSS software (version 28, IBM SPSS Statistics, Orchard Road Armonk, NY, USA), ensuring objective interpretation and reproducibility of the results.

## 3. Results and Discussion

### 3.1. Screening and Physical Compatibility of Lipid Excipients

The initial visual screening of excipients was included as a preliminary qualitative step to rapidly identify candidate solid and liquid lipids capable of solubilizing KTZ at elevated temperatures and forming physically homogeneous systems. This step allowed the exclusion of clearly unsuitable excipients before proceeding to the more time-consuming and quantitative saturation solubility studies, thereby streamlining formulation development and minimizing unnecessary experimental runs. The selection of suitable lipids is essential for the successful preparation of NLCs loaded with poorly soluble drugs like KTZ, since this selection process directly affects drug encapsulation and the EE of NLCs. Furthermore, high EE can also be attained by adding liquid lipids to form incomplete lattices and load more drug molecules [18,22].

The selection of solid and liquid lipids subjected to screening and compatibility studies was guided by the physicochemical properties of KTZ, a highly lipophilic BCS class II drug (log P ≈ 4.74) with extremely low aqueous solubility. Such compounds exhibit preferential partitioning into lipidic matrices, making lipid-based nanocarriers particularly suitable for their topical delivery. Accordingly, a representative set of pharmaceutically accepted solid and liquid lipids was chosen to provide diversity in chemical composition, melting behaviour, and hydrophobicity. Fatty acid esters and glyceride-based lipids, such as Precirol^®^ ATO 5 and oleic-acid-based systems, were included due to their reported high solubilization capacity for lipophilic drugs and their ability to form imperfect crystalline matrices when combined, a key requirement for nanostructured lipid carriers. Liquid lipids were further selected based on their known role in disrupting lipid crystallinity, enhancing drug accommodation, and improving skin permeation.

The preliminary visual screening served to exclude lipid excipients with negligible affinity for KTZ, while the subsequent physical compatibility studies ensured homogeneity and absence of phase separation upon solidification. This stepwise approach enabled rational selection of excipients capable of forming stable NLC matrices with high drug loading potential, consistent with previous reports on lipid-based delivery systems for azole antifungals and other lipophilic drugs [11,18,21,23].

KTZ showed higher solubility in oleic acid, soybean oil, castor oil, and Precirol^®^ ATO 5 compared with the other screened lipids (Table 3). Thus, these four lipids were the candidates for the saturation solubility studies. Furthermore, no separation was visually observed in the congealed matrix of solid and liquid lipids within two days at room temperature with all tested solid and liquid lipid ratios.

### 3.2. Saturation Solubility Studies

The maximum solubility of KTZ in each of the four selected oils was determined through saturation solubility experiments. The temperature of 80 °C used in the saturation solubility studies of ketoconazole in solid lipids was selected based on the melting point characteristics of the solid lipid excipients, rather than on the thermal properties of the API itself. KTZ is thermally stable at this temperature range, while the selected solid lipids (e.g., Precirol^®^ ATO 5 and other screened lipids) require heating to temperatures between 65 and 75 °C to achieve complete melting and formation of a homogeneous lipid phase. Conducting solubility studies at 80 °C ensured that the solid lipids remained fully molten throughout the equilibration period, enabling accurate assessment of the maximum solubilization capacity of KTZ within the lipid melt under conditions representative of the NLC preparation process. The results showed that 205.4 ± 3.5, 95.7 ± 1.8, 11.2 ± 0.7, and 170.3 ± 1.2 mg of KTZ were soluble in 1.0 g of oleic acid, castor oil, soybean oil, and Precirol^®^ ATO 5, respectively (mean ± SD, N = 3). The results demonstrate that KTZ showed superior solubility in oleic acid compared to the other evaluated oils. Based on this observation, oleic acid along with Precirol^®^ ATO 5 was chosen as the oil phase for formulating the NLCs.

### 3.3. Effect of Formulation Variables on PS of NLCs

After data analysis using DesignExpert^®^ software, it was evident that all four independent variables affected the PS of KTZ-NLCs significantly. PS obtained from the 16 experimental runs ranged from 212.0 ± 8.7 to 374.2 ± 10.4 nm. Analysis of variance (ANOVA) revealed that the selected factorial model was significant (*p* < 0.0001). The model F-value of 24.67 further confirmed its significance. Among the evaluated parameters, only factors A, B, C, and D were identified as significant model terms. The predicted R^2^ value showed good agreement with the adjusted R^2^ value (difference < 0.2), indicating strong model predictability. The mean PS of KTZ-NLCs was significantly increased with the increase in Precirol^®^ ATO 5 (A), oleic acid (B), and Span^®^ 80 concentrations (C) and the decrease in Tween^®^ 80 concentrations (D), as shown in Figure S1. The various correlation data for fitting the selected factorial model of the PS response are shown in Table 4. In the polynomial equations, a positive coefficient indicates that the response increases as the corresponding factor level increases. Conversely, a negative coefficient signifies an inverse relationship, whereby the response decreases as the factor level increases. Moreover, the magnitude of each coefficient reflects the extent to which its corresponding factor influences the response. The following equation represents the final equation for predicting PS in terms of coded factors:

R_1_ (PS) = +268.75 + 26.29 × A + 16.87 × B + 19.10 × C − 10.16 × D

The observed influence of formulation variables on PS can be rationalized by considering the physicochemical properties of the excipients and KTZ. An increase in the solid lipid content within the NLCs has been reported to increase PS, which is attributed to the higher lipid density introduced by the additional solid lipid [24]. The solid lipid Precirol^®^ ATO 5 is composed mainly of long-chain fatty acid esters (palmitic and stearic acid derivatives), characterized by relatively high melting points and a high degree of crystalline order. Increasing the concentration of such long-chain lipids elevates melt viscosity and lipid phase rigidity, thereby reducing droplet disruption efficiency during homogenization and sonication, which ultimately results in larger particle sizes.

The concentration of the liquid lipid also exerts a significant influence on the PS of the NLCs, a finding that may be attributed to the expansion of the crystalline matrix resulting from the incorporation of the liquid lipid. Increasing the proportion of oleic acid, a long-chain unsaturated fatty acid with a cis-double bond, leads to expansion of the lipid matrix and reduced packing efficiency. While this structural disorder benefits drug accommodation, it also increases the effective lipid volume and viscosity of the dispersed phase, contributing to particle growth when emulsification energy is constant. As the total lipid amount increases, emulsifying efficiency decreases, resulting in increased surface tension and the formation of larger particles. These observations were consistent with several earlier reports [25,26,27].

Surfactant composition further played a critical role in governing particle size through interfacial stabilization. Span^®^ 80 (low HLB, lipophilic sorbitan monooleate) preferentially partitions into the lipid phase and reduces interfacial curvature, which can promote formation of larger droplets when used at higher levels. Moreover, the increase in the % of Span^®^ 80 in the total mixture of surfactants decreased the Hydrophilic Lipophilic Balance (HLB) value of the surfactant mixture and consequently increased PS significantly (*p* < 0.05). In contrast, Tween^®^ 80 (high HLB, polyoxyethylene sorbitan monooleate) enhances aqueous solubilization and lowers interfacial tension more efficiently, facilitating droplet breakup under shear stress and producing smaller particles [28]. Thus, increasing the concentration of Tween^®^ 80 led to a marked decrease in PS, likely due to the reduction in interfacial tension and surface free energy under high-shear homogenization conditions [18,29].

Additionally, KTZ itself is a highly lipophilic molecule containing aromatic rings and heterocyclic nitrogen atoms, which favor strong hydrophobic interactions with long-chain lipids. Such drug–lipid affinity can influence lipid matrix organization and, at higher lipid concentrations, contribute indirectly to PS enlargement. Overall, the PS trends observed in this study reflect a balance between lipid chain length, surfactant HLB, interfacial tension, and lipid–drug interactions, consistent with previously reported NLC formulation studies.

### 3.4. Effect of Formulation Variables on ZP of NLCs

Only one independent variable was found to significantly (*p* < 0.05) influence the ZP of KTZ-NLCs. The ZP values across the 16 experimental runs ranged from −22.1 ± 0.2 to −25.7 ± 0.4 mV. Statistical analysis using ANOVA confirmed that the model was highly significant (*p* < 0.0001), with an F-value of 34.47. Among the tested factors, oleic acid (factor B) was identified as the only significant contributor to ZP variation, as illustrated in Figure S2. Additionally, the predicted R^2^ value closely matched the adjusted R^2^ (difference < 0.2), indicating strong model reliability and predictive accuracy. The final equation describing ZP in terms of coded factors is presented below.

R_2_ = −22.96 − 1.12 × B

The mean ZP values of KTZ-NLCs increased in magnitude with higher oleic acid content, indicating a greater accumulation of negative charges on the particle surface. Since both the sign and magnitude of ZP reflect the type and density of surface charge, this trend suggests enhanced surface ionization with increasing oleic acid levels. This behavior is consistent with previous reports and can be attributed to the presence of ionizable carboxyl groups in oleic acid, which contribute to the overall negative charge of the formulation [30,31].

### 3.5. Optimization

PS is a key factor for a successful nanoparticulate formulation since it affects the quality features of these nanocarriers, such as drug loading, release profile, in vivo biodistribution, and biological fate [15]. Smaller PS has a larger surface area-to-volume ratio and can entrap less drug and release the drug at a faster rate. Moreover, PS is a critical prerequisite parameter for nanocarriers to cross biological barriers [15]. However, ZP is a well-established parameter to examine the long-term stability of the nanocarriers. Based on the literature review, topical NLC formulations with PS ≤ 300 nm improved drug penetration through skin layers [32]. In addition, NLC preparations should have a ZP value of ± 30 mV for maximal physical stability [33]. Following the analysis of the responses and the establishment of robust regression models, an optimization step was carried out to determine the optimal levels of the formulation factors. The criteria applied to the independent variables and responses during the optimization process are summarized in Table 5. To obtain the desired responses within the 95% confidence interval (CI), the software identified a single optimal solution, which closely matched the composition of the F11 formulation. The optimized solution is illustrated through cube plots (Figure S3). A validation study was carried out in triplicate to assess the agreement between experimental and predicted values of PS and ZP, as summarized in Table 6. The observed values were found to fall within the 95% CI of the predicted results, confirming the reliability of the model.

### 3.6. Preparation of KTZ-NLCs and KTZ-NLCs-C

The type and the concentration of excipients used in the formulation development process are critical in terms of formulation safety. All formulation excipients were evaluated at concentrations falling within the inactive-ingredient limits approved by the United States Food and Drug Administration (USFDA) for topical drug products, as summarized in Table 7. It is well-established that a 1–5% *w*/*v* surfactant concentration is required for NLC preparation [34]. In addition, a mixture of surfactants with an adjusted HLB value greater than 10 was reported to produce stable NLC dispersions [34]. Moreover, nonionic surfactants such as Tween^®^ 80 and Span^®^ 80 are reported to have lower irritation potential and toxicity compared to ionic surfactants [35,36].

Carbomer copolymers (types A, B, and C) and homopolymers (types A, B, and C) are widely incorporated into FDA-approved topical formulations. Among these, Carbopol^®^ 940 NF is recognized as an effective thickening agent for clear aqueous and hydroalcoholic gels, offering high viscosity (40,000–60,000 cP at 0.5% *w*/*w* and pH 7.5) within a concentration range of 0.5–3.0% *w*/*w*. Accordingly, Carbopol^®^ 940 NF was incorporated into the optimized KTZ-NLC formulation (F11) at 0.5% *w*/*w* to obtain the KTZ-NLCs-C system. Notably, this polymer remains in a liquid state under acidic conditions and undergoes a sol–gel transition to form a viscoelastic gel when the pH exceeds 5.5 [37]. Thus, Carbopol^®^ 940 was selected in this study to improve the adhesion of the formulation to the skin surface. The composition of all prepared KTZ-NLCs is presented in Table 8. All prepared NLC formulations were loaded with 1% *w*/*v* of KTZ.

### 3.7. Measurement of PS, PDI, and ZP

Prior to DLS analysis, samples were diluted with filtered Milli-Q water to minimize multiple scattering effects, which is a standard requirement for accurate PS determination. Although excessive dilution may potentially influence surfactant distribution at the particle surface, the formulations showed consistent PS, low PDI, and good reproducibility across replicate measurements, indicating that dilution did not induce significant aggregation or instability.

PS, PDI, and ZP of all prepared formulations are presented in Table 2 and Figure S4. The size of the nanocarrier can significantly influence both the extent and depth of drug deposition across the skin layers [38]. For the topical route, a PS of less than 500 nm is preferred for dermal drug delivery to penetrate the skin epithelium [39]. All NLC formulations showed PS below 400 nm, aiming for topical application. PDI values reveal the width of PS distribution, while a PDI > 0.5 indicates a broad distribution [40]. ZP is an important parameter that can predict the physical stability of nanodispersions. Higher positive or negative ZP values enhance the stability of colloidal dispersions by minimizing particle aggregation through electrostatic repulsion among similarly charged NLC particles. A ZP of more than ±30 mV provides good physical stability and becomes excellent when ZP values approach ±60 mV [41]. However, a ZP value of >±20 could provide good physical stability [41].

The incorporation of Carbopol^®^ 940 NF markedly altered the ZP, shifting it from −23.5 ± 0.5 in the optimized NLCs (F11) to −35.2 ± 0.5 in the KTZ-NLCs-C formulation. This change can be attributed to the presence of negatively charged carboxyl groups along the polymer backbone. The notable increase in negative ZP suggests that Carbopol^®^ 940 NF is adsorbed onto the surface of the NLCs [20]. The significant increase (*p* < 0.05) in PS from 227.7 ± 5.5 for the F11 formulation to 285.9 ± 3.0 for the mucoadhesive formulation also supports the surface adsorption of Carbopol^®^ 940 NF [23]. Moreover, the PDI of the mucoadhesive formulation increased simultaneously with PS, from 0.27 ± 0.03 for the F11 formulation to 0.43 ± 0.05.

It is important to note that the developed system represents a liquid dispersion in which lipid nanoparticles exist in a hydrated and colloidal state. Dynamic light scattering (DLS) therefore provides the hydrodynamic diameter of particles in their native dispersion environment and is widely considered an appropriate technique for size characterization of such systems.

However, DLS does not provide direct information on particle morphology, internal structure, or lipid crystallinity. Complementary techniques such as cryogenic transmission electron microscopy (cryo-TEM) would allow visualization of nanoparticles in their native hydrated state, while differential scanning calorimetry (DSC) could provide insight into lipid crystallinity and matrix organization. Fourier-transform infrared spectroscopy (FTIR) may also be used to evaluate potential drug–excipient interactions. The absence of these techniques represents a limitation of the current study and will be addressed in future investigations.

### 3.8. KTZ Drug Content and EE (%)

According to the United States Pharmacopeia (USP), ±10% variation in the limits of drug content (assay) from the label claim (100%) is proposed to account for the variability in manufacturing and shelf-life stability. Such variations in drug content are unlikely to negatively affect the intended therapeutic efficacy or the product’s toxicity profile (USP). Drug content and EE are considered good indicators of the chemical stability of all nanodispersions, including NLCs. The KTZ content in the NLC formulations ranged from 96.6 ± 2.1 to 104.9 ± 2.1% as depicted in Figure S5, with a % drug content value of 99.7 ± 0.1 for the optimized KTZ-NLC formulation (F11). The KTZ-NLCs-C formulation showed similar drug content values (98.6 ± 2.3) to the optimized NLC formulation.

It has been reported that combining solid and liquid lipids creates a more disordered matrix structure, introducing imperfections within the crystal lattice and generating additional spaces capable of accommodating more drug molecules. This structural disruption enhances both entrapment efficiency and drug loading capacity [42]. The % EE of NLC formulations ranged from 91.1 ± 0.1 to 99.9 ± 0.0 as depicted in Figure S6, with a % EE value of 97.4 ± 0.1 for the optimized KTZ-NLC formulation. The higher encapsulation efficiency may result from the loosened or imperfect lipid matrix formed by the interaction between oleic acid and the palmitic (C16) and stearic (C18) acid esters in Precirol^®^ ATO 5. This less compact structure creates additional space within the matrix, allowing more KTZ molecules to be incorporated [18]. It is worth mentioning that the mucoadhesive formulations showed similar EE (97.5 ± 3.0) to the optimized NLC formulation.

### 3.9. pH Measurement

The pH values for all KTZ-NLC formulations are shown in Figure S7. There is a high level of agreement that topically applied products should be acidified to a pH in the range of 4.0 to 6.0 [39]. The mildly acidic pH of the skin plays a key role in regulating stratum corneum homeostasis and maintaining barrier integrity and permeability [39]. All prepared NLC batches showed a narrow range of pH values (4.36 ± 0.01–4.68 ± 0.01), which falls within the ideal range for topical formulations. The significant decrease in pH from 4.34 ± 0.01 (F11) to 3.32 ± 0.01 (KTZ-NLCs-M) could be due to the carboxylic acid groups of Carbopol^®^ 940 NF.

### 3.10. Viscosity

**Figure 1:**
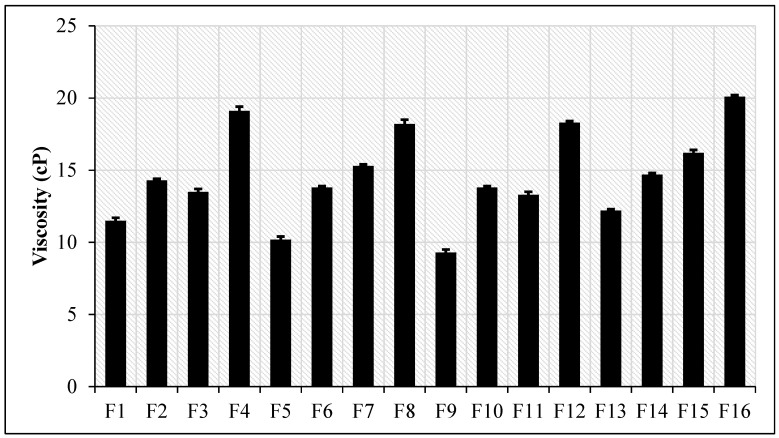
Viscosity (cP) of all Ketoconazole-loaded NLCs at 25 °C and 10 rpm (mean ± SD, N = 3).

The viscosity of a fluid is a measurement of its resistance to flow; the higher the viscosity, the more resistant the fluid. The rheological properties of dermal products are of great importance when designing a new formulation. Viscosity not only affects product spreadability and skin sensation but also could affect the drug permeation through the skin [43]. Drug penetration has been shown to decrease as the viscosity of dermal formulations increases [43]. The viscosity measurements for all formulations are illustrated in Figure 1. The viscosity values ranged from 9.3 ± 0.2 to 20.1 ± 0.1 cP. The viscosity of the optimized NLC formulation (F11) increased markedly from 13.3 ± 0.2 cP to 32.5 ± 0.4 cP following the incorporation of Carbopol^®^ 940 NF (KTZ-NLCs-C). This increase was statistically significant (*p* < 0.05) and aligned with expectations based on the polymer’s rheological behavior.

### 3.11. In Vitro Release Testing

**Figure 2:**
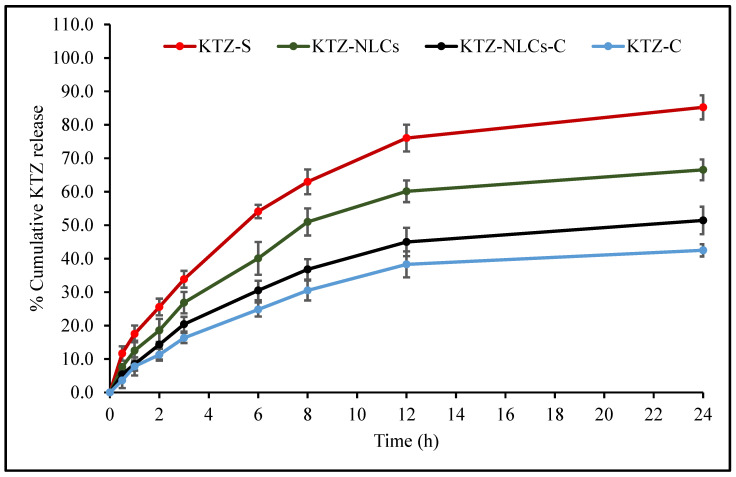
In vitro release of ketoconazole from KTZ-S, KTZ-C, optimized KTZ-NLCs, and KTZ-NLCs-C formulations (mean ± SD, N = 3).

The cumulative percentage of KTZ released from each formulation was plotted over time, and the corresponding in vitro release profiles are presented in Figure 2. After 24 h, the extent of drug release followed the order: KTZ suspension (88.2 ± 3.1%) > optimized NLCs (66.5 ± 4.1%) > mucoadhesive NLCs (51.4 ± 4.1%) > KTZ cream (42.5 ± 1.8%). This trend may be attributed to the highly lipophilic nature of KTZ (log P ≈ 4.74), which limits its aqueous solubility [44]. The controlled release profile of the drug from the optimized NLCs and its corresponding mucoadhesive formulation could be attributed to the lipophilic nature of KTZ that is deeply entrapped in the crystalline matrix of the nanoparticles. Therefore, the drug has a longer diffusion path to reach the dissolution medium, consistent with earlier reported studies [45]. However, the KTZ-NLCs-C formulation, containing Carbopol^®^ 940 NF, showed a slower drug release compared to the optimized KTZ-NLCs (F11) formulation. This is not surprising, as the mucoadhesive NLC formulation showed higher viscosity compared to the optimized NLC formulation; therefore, drug diffusion was even slower. Moreover, the KTZ release was slower from both NLC formulations compared to the suspension formulation. This could be due to the fact that the drug molecules are dispersed in the solid crystalline lipid matrix in the case of the NLC formulations.

The kinetic model exhibiting the highest coefficient of determination (R^2^) was selected as the most appropriate to describe the drug release behavior of the formulation. The R^2^ values corresponding to each model are summarized in Table 9. The maximum coefficient of determination (R^2^) was obtained with the Korsmeyer–Peppas model for both the optimized NLC formulation and its corresponding mucoadhesive system, indicating that this model best describes their drug release behavior. The optimized NLC formulations showed the highest R^2^ values for the Korsmeyer–Peppas model (0.9984). The slope (n) of the Korsmeyer–Peppas model for the optimized KTZ-NLCs and lead KTZ-NLCs-C formulations was 0.63 and 0.59, respectively, which indicated a non-Fickian (0.45 < n < 0.89) or anomalous drug release profile controlled by erosion and diffusion mechanisms. Previous studies have reported similar release profiles for lipid nanoparticles and for hydrogels incorporating these nanoparticles [18].

### 3.12. Ex Vivo Permeation and Deposition Testing

**Figure 3:**
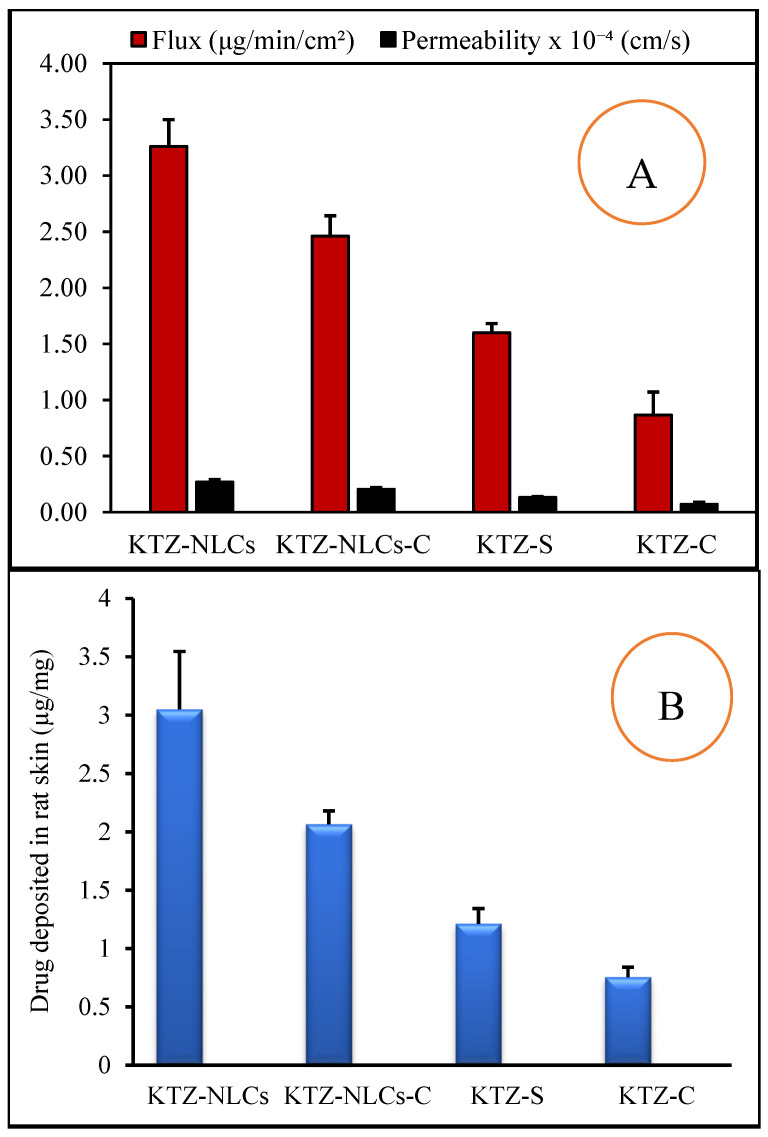
(**A**) Transdermal flux and permeability coefficients and (**B**) skin deposition results of Ketoconazole from optimized KTZ-NLCs, KTZ-NLCs-C, suspension and cream formulations through rat skin (mean ± SD, N = 3).

Ex vivo drug permeation and deposition studies through the skin can be utilized to predict percutaneous absorption in humans [46]. The ex vivo permeation profiles of all tested formulations are presented in Figure 3A,B. The flux of KTZ from the tested formulations was in the following descending order: KTZ-NLCs (3.26 ± 0.24 μg/min/cm^2^), KTZ-NLCs-C (2.46 ± 0.18 μg/min/cm^2^), KTZ suspension (KTZ-S, 1.60 ± 0.08 μg/min/cm^2^), and KTZ cream (KTZ-C, 0.87 ± 0.12 μg/min/cm^2^). The transdermal permeability coefficients of KTZ-NLCs and KTZ-NLCs-C formulations were 0.27 ± 0.02 and 0.21 ± 0.02 × 10^−4^ cm/min, respectively. The ex vivo transdermal flux and permeability coefficient of KTZ from the optimized KTZ-NLC formulation were approximately 3.7-fold greater than those achieved with the marketed cream formulation. Similarly, the ex vivo transdermal flux and permeability coefficient of KTZ from the lead KTZ-NLCs-C formulation were approximately 2.8-fold greater than those achieved with the marketed cream formulation. The reason for the enhanced transdermal penetration from both NLC-based formulations could be attributed to the adhesive and occlusive nature of these nanoparticles, which improves skin hydration and thus drug permeation [47,48]. Moreover, oleic acid acts as a permeation enhancer by inducing the formation of lacunae and disrupting the intercellular lipid bilayers within the stratum corneum, thereby facilitating increased drug penetration across this protective barrier [49,50].

However, the ex vivo transdermal flux and permeability coefficient of KTZ from the optimized KTZ-NLC formulation were significantly (*p* < 0.05) higher than those observed with its corresponding mucoadhesive Carbopol^®^ 940-containing formulation (KTZ-NLCs-C). The reason for the significantly lower ex vivo skin permeation potential of the mucoadhesive formulation, as compared to the optimized KTZ-NLCs, could be attributed to the slow permeation of KTZ due to the higher viscosity of the formulation. Moreover, both NLC-based formulations demonstrated notably greater skin deposition than the control formulations, which may be explained by the interaction, adsorption, and possible fusion of the lipid matrix with the skin surface. The developed NLC system was rationally designed to enhance localized drug delivery while minimizing systemic absorption, which is a critical requirement for topical antifungal therapy. This balance is achieved through multiple formulation-related factors. The high lipophilicity of KTZ promotes its preferential partitioning into the stratum corneum, limiting systemic diffusion. In addition, the lipid-based nanocarrier forms an occlusive and adhesive film on the skin surface, enhancing hydration and facilitating drug retention within superficial skin layers. The incorporation of Carbopol^®^ 940 NF further increases formulation viscosity and residence time, thereby promoting local retention and reducing drug diffusion away from the application site. This is supported by the ex vivo findings, where enhanced skin deposition and controlled permeation were observed, with the mucoadhesive formulation exhibiting a slightly reduced flux due to increased viscosity. Collectively, these results indicate that the developed system effectively improves dermal drug localization while limiting systemic exposure.

### 3.13. Skin Irritation Studies

Skin irritation was evaluated in rats using KTZ-S and the marketed cream as controls, formalin (0.8% *w*/*v*) as a positive control, and an untreated group as a negative control. The results (Table 10) showed that none of the NLC-based formulations caused any visible changes in skin color or morphology during the 24 h observation period. In contrast, KTZ-S induced erythema and edema, while the marketed cream produced erythema at the same time point. These findings suggest that the NLC-based formulations are safe and suitable for topical application.

### 3.14. In Vitro Antifungal Activity

**Figure 4:**
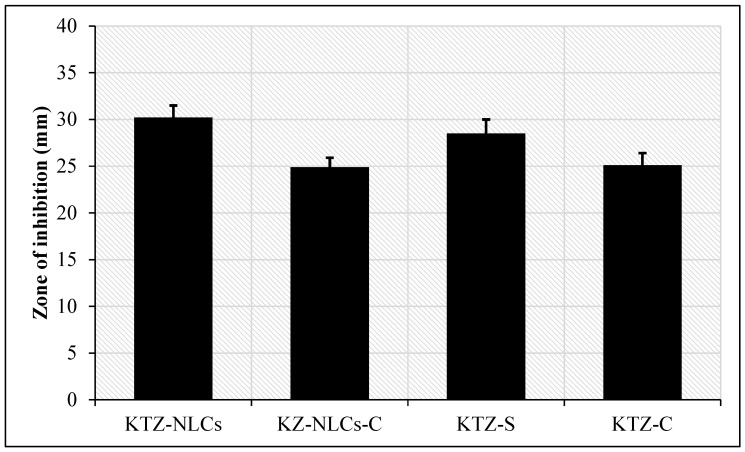
In vitro antifungal susceptibility testing of the optimized KTZ-NLCs, KTZ-NLCs-C, Ketoconazole suspension, and ketoconazole cream formulations against *Candida albicans* (mean ± SD, N = 4). Statistical analysis was performed using one-way ANOVA followed by Tukey’s post hoc test (*p* < 0.05).

The antifungal activity results were statistically evaluated using one-way ANOVA, followed by Tukey’s post hoc test to assess significant differences between the formulations. All experiments were performed in quadruplicate (N = 4), and results are expressed as mean ± SD. The cup plate method reflects a combined effect of intrinsic antifungal activity and the diffusion behavior of the formulation within the agar matrix. Therefore, broth microdilution MIC assays represent an important next step and will be the focus of future investigations to complement the current formulation-oriented findings. In vitro antifungal susceptibility testing showed that KTZ demonstrated improved antifungal efficacy when incorporated into the NLC and NLC-C formulations, as shown in Figure 4. An in-house-prepared KTZ suspension (KTZ-S) was also used in the current study because the cream formulation is too thick and the drug may not diffuse well during the test. The antifungal efficacy of the optimized KTZ-NLCs against *Candida albicans* was significantly (*p* < 0.05) greater than that of the marketed cream and KTZ suspension. The enhanced antifungal activity of KTZ-NLCs may be attributed to their large specific surface area, which facilitates close interaction with the fungal cell membrane. This effect could also be linked to their ability to interact with ergosterol-rich structures in fungal hyphae. In comparison, the antifungal effect of KTZ-NLCs-C against Candida albicans was lower than that of the KTZ suspension and the optimized KTZ-NLC formulation and was comparable to the marketed cream. This was expected, as the lead NLC formulation is more viscous than the KTZ suspension and the optimized KTZ-NLC formulation. It is worth mentioning that the negative control vehicles used in the study did not show activity against *Candida albicans.* In conclusion, the KTZ-based NLC and NLC-C formulations showed promising antifungal activity. Furthermore, KTZ shows improved antifungal efficacy than the isolated drug when it is loaded inside NLCs, since it would facilitate the penetration or interaction of KTZ with the fungal cell surface.

### 3.15. Physicochemical Stability

**Figure 5:**
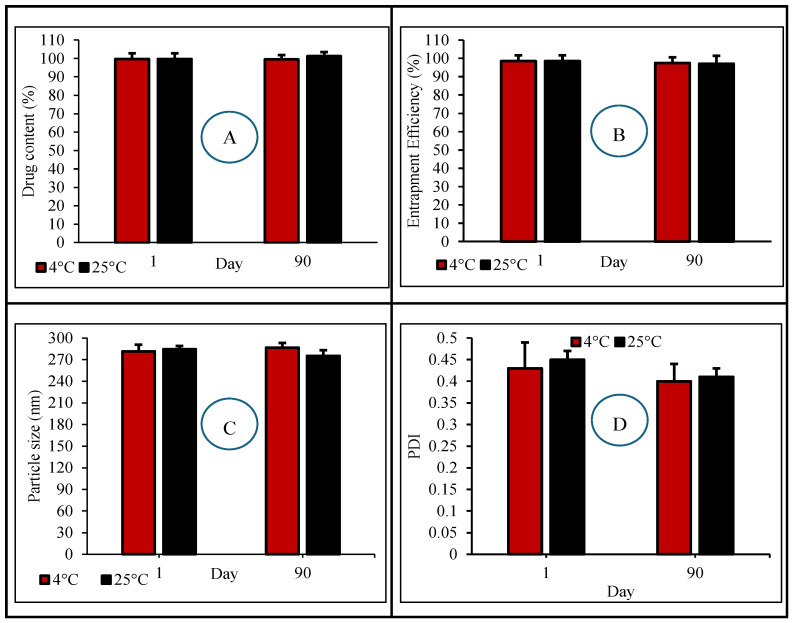
(**A**) Ketoconazole content, (**B**) Entrapment efficiency, (**C**) Particle size, and (**D**) Polydispersity index of the lead mucoadhesive KTZ-NLCs-C formulation over 90-day storage at 4 and 25 °C (mean ± SD, N = 3).

The physicochemical stability of the optimized formulation (KTZ-NLCs-C) was assessed after storage under refrigerated and room temperature conditions for 90 days. No visible signs of instability, such as cracking, were observed throughout the study period. Additionally, only slight and statistically insignificant changes (*p* > 0.05) were detected in PS, ZP, PDI, pH, viscosity, drug content, and EE, indicating good stability, as illustrated in Figure 5A–D and Figure S8.

## 4. Conclusions

KTZ-loaded NLCs and mucoadhesive NLC formulations were successfully developed and optimized using Carbopol^®^ 940 NF as a gelling agent. The ex vivo studies demonstrated improved skin permeability and flux when compared to the marketed cream. In addition, NLC-based formulations were suitable for topical applications during skin irritation testing studies. Furthermore, the formulations were physiochemically stable for 90 days (last time point assessed) at both tested storage conditions. Overall, the NLC and NLC-C prepared in this project are suitable for bioavailability enhancement of KTZ through the skin. Thus, the NLCs combined with a gelling agent could serve as an efficient drug delivery platform for the different topical applications of KTZ.

## Figures and Tables

**Table 1:** Independent and dependent variables (factors) along with their coded levels employed in the full factorial experimental design of ketoconazole nanolipid carriers.

Factors	Coded Levels	
Independent variables (% *w*/*v*)	Level −1	Level +1
A: Solid lipid	3.0	4.0
B: Oil	1.0	2.0
C: Span^®^ 80	0.5	1.0
D: Tween^®^ 80	2.0	3.0
Dependent variables		
R_1_: Particle size (PS, nm)		
R_2:_ Zeta potential (ZP, mV)		

**Table 2:** Full factorial design parameters for different experimental runs, along with the observed values of particle size and zeta potential of ketoconazole nanolipid carriers.

Run	Assigned Independent Variables				Actual Independent Variables (% *w*/*v*)				Response	
	A	B	C	D	Precirol^®^ ATO 5	Oil (Oleic acid)	Span^®^ 80	Tween^®^ 80	PS (nm)	ZP (mV)
1	−1	−1	−1	−1	3.0	1.0	0.5	2.0	227.5 ± 6.7	−22.9 ± 0.7
2	+1	−1	−1	−1	4.0	1.0	0.5	2.0	269.5 ± 10.4	−21.8 ± 0.4
3	−1	+1	−1	−1	3.0	2.0	0.5	2.0	252.9 ± 4.8	−23.8 ± 0.5
4	+1	+1	−1	−1	4.0	2.0	0.5	2.0	286.8 ± 5.5	−25.7 ± 0.4
5	−1	−1	+1	−1	3.0	1.0	1.0	2.0	245.9 ± 10.8	−21.2 ± 0.3
6	+1	−1	+1	−1	4.0	1.0	1.0	2.0	309.1 ± 7.4	−21.7 ± 0.4
7	−1	+1	+1	−1	3.0	2.0	1.0	2.0	265.4 ± 22.9	−23.3 ± 0.6
8	+1	+1	+1	−1	4.0	2.0	1.0	2.0	374.2 ± 10.4	−23.4 ± 0.7
9	−1	−1	−1	+1	3.0	1.0	0.5	3.0	212.0 ± 8.7	−21.5 ± 0.5
10	+1	−1	−1	+1	4.0	1.0	0.5	3.0	240.9 ± 10.1	−21.2 ± 1.2
11	−1	+1	−1	+1	3.0	2.0	0.5	3.0	227.7 ± 5.5	−23.5 ± 0.5
12	+1	+1	−1	+1	4.0	2.0	0.5	3.0	279.9 ± 6.7	−24.9 ± 0.7
13	−1	−1	+1	+1	3.0	1.0	1.0	3.0	231.4 ± 3.2	−22.1 ± 0.2
14	+1	−1	+1	+1	4.0	1.0	1.0	3.0	278.7 ± 5.9	−22.3 ± 0.3
15	−1	+1	+1	+1	3.0	2.0	1.0	3.0	276.9 ± 7.2	−23.3 ± 0.9
16	+1	+1	+1	+1	4.0	2.0	1.0	3.0	321.2 ± 6.6	−24.7 ± 0.7

**Table 3:** Visual screening of lipids for Ketoconazole-loaded nanolipid carriers (KTZ-NLCs).

Oil	Solubility	Solid Lipid	Solubility
Soybean oil	(√)	Gelucire^TM^ 50/13	(×)
Cottonseed oil	(×)	Gelucire^TM^ 43/01	(×)
Sesame oil	(×)	Gelucire^TM^ 44/14	(×)
Oleic acid	(√)	Precirol^®^ ATO 5	(√)
Isopropyl myristate	(×)	Softisan^®^ 154	(×)
Maisine^®^ CC	(×)	Imwitor^®^ 900 K	(×)
Castor oil	(√)	Imwitor^®^ 960 K	(×)
Labrasol^®^	(×)	Geleol^TM^	(×)

(√): KTZ dissolved in the tested lipid; (×): KTZ did not dissolve in the tested lipid.

**Table 4:** Analysis of variance (ANOVA) for the regression coefficients of the developed models predicting particle size and zeta potential.

Response	F Value	*p*-Value	R^2^	Predicted R^2^	Adjusted R^2^	Adequate Precision *
R_1_: PS	24.67	0.0001	0.8997	0.7878	0.8632	16.935
R_2_: ZP	34.47	0.0001	0.7112	0.6227	0.6905	8.303

* Adeq Precision evaluates the signal-to-noise ratio of the model, with values exceeding 4 generally considered indicative of an adequate signal. Such a ratio suggests that the model possesses sufficient discriminatory capability and can therefore be reliably used to explore and navigate the design space.

**Table 5:** The criteria applied to the variables and responses during the optimization process.

Variable	Goal	Lower Limit	Upper Limit
Precirol^®^ ATO	is in range	3.0% *w*/*v*	4.0% *w*/*v*
Oleic acid	is in range	1.0% *w*/*v*	2.0% *w*/*v*
Span^®^ 80	is in range	0.5% *w*/*v*	1.0% *w*/*v*
Tween^®^ 80	is in range	2.0% *w*/*v*	3.0% *w*/*v*
Particle size (nm)	target = 200 nm	100 nm	374.2 nm
Zeta potential (mV)	Minimize	−21.2 mV	−30.0 mV

**Table 6:** Results of validation trials of the proposed solution (N = 4).

Response	Predicted Value	Results of Validation Trials	95% CI (Low for Mean)	95% CI (High for Mean)
PS (nm)	230.1	228.6	211.2	248.9
ZP (mV)	−24.1	−23.8	−24.7	−23.5

**Table 7:** Excipients used in the preparation of KTZ-NLCs and KTZ-NLCs-C formulations as per the US Food and Drug Administration inactive ingredient database.

Inactive Ingredient	Topical Dosage Form	Maximum Potency Per Unit Dose (% *w*/*w*)	Maximum Daily Exposure (MDE, mg)
Precirol^®^ ATO 5	NA		100 *
Oleic acid	Cream	25	
	Gel, Metered		88
	Solution	7.4	
Span^®^ 80	Cream	3.5	
	Cream, Augmented	0.2	
	Emulsion	2.5	
	Gel	1	
	Lotion	7	
	Ointment	NA	
	Spray	0.25	
Tween^®^ 80	Aerosol, Foam	0.98	
	Cream		4
	Emulsion	2.5	
	Gel	8.5	
	Lotion	15	
	Ointment	0.1	
Carbopol^®^ 940 NF	Cream		103
	Cream, Augmented		20
	Emulsion		6
	Gel		85
	Lotion		300
	Ointment, Augmented		23

* The reported value is for an oral dosage form.

**Table 8:** The composition of different KTZ-NLCs and KTZ-NLCs-C formulations.

Code	Precirol^®^ ATO 5 (mg)	Oleic Acid (mg)	Span^®^ 80 (mg)	Tween^®^ 80 (mg)	KTZ (mg)	Carbopol^®^ 940 (mg)	Water up to (mL)
F1	300	100	50	200	100	-	10
F2	400	100	50	200	100	-	10
F3	300	200	50	200	100	-	10
F4	400	200	50	200	100	-	10
F5	300	100	100	200	100	-	10
F6	400	100	100	200	100	-	10
F7	300	200	100	200	100	-	10
F8	400	200	100	200	100	-	10
F9	300	100	50	300	100	-	10
F10	400	100	50	300	100	-	10
F11	300	200	50	300	100	-	10
F12	400	200	50	300	100	-	10
F13	300	100	100	300	100	-	10
F14	400	100	100	300	100	-	10
F15	300	200	100	300	100	-	10
F16	400	200	100	300	100	-	10
KTZ-NLCs-C	300	200	50	300	100	50	10

**Table 9:** Analysis of regression for the release profiles of ketoconazole from the optimized nanolipid carriers and their respective mucoadhesive nanolipid carrier formulations (mean ± SD, N = 4).

Formulations	Zero Order	First Order	Higuchi	Korsmeyer-Peppas	
	Q_0_ − Q = k·t	ln Q = k·t	Q_0_ − Q = k·t^1/2^	log (Q_0_ − Q) = n log t + log k	
	R^2^	R^2^	R^2^	R^2^	n
KTZ-S	0.5349	0.9748	0.9599	0.9984	0.643
Optimized NLCs (F11)	0.5654	0.9033	0.9534	0.9942	0.630
KTZ-NLCs-C	0.6095	0.8375	0.9547	0.9601	0.585
KTZ-C	0.6215	0.8010	0.9587	0.9589	0.493

Q_0_ represents the initial drug content at time t_o_, and Q represents the drug content remaining at time t; Zero-order model: % drug released vs. time; First-order model: Amount of drug remaining vs. time; Higuchi model: % drug released vs. square root of time; Korsmeyer–Peppas model: log % drug released vs. log time.

**Table 10:** Effect of the optimized KTZ-NLCs and KTZ-NLCs-C formulations on male albino rat skin during 24 h of topical application (N = 4).

Group	Formulation	Erythema Score			Edema Score		
		6 h	12 h	24 h	6 h	12 h	24 h
I	Negative Control	0	0	0	0	0	0
II	KTZ-NLCs	0	0	0	0	0	0
III	KTZ-NLCs-C	0	0	0	0	0	0
VI	KTZ-S	0	0	1	0	0	2
V	KTZ-C	0	0	1	0	0	0
VI	Formalin (0.8% *w*/*v*)	0	1	2	1	2	3

Erythema scale: 0 = none, 1 = slight, and 2 = well-defined. Edema scale: 0 = none, 1 = slight, 2 = well-defined, and 3 = moderate.

## Data Availability

The original contributions presented in this study are included in the article/Supplementary Materials. Further inquiries can be directed towards the corresponding author.

## Supplementary Materials

The following supporting information can be downloaded at https://www.mdpi.com/article/10.3390/pharmaceutics18060753/s1, Figure S1: Response surface plots illustrating the effects of (A) solid lipid and liquid lipid concentrations, and (B) Span^®^ 80 and Tween^®^ 80 levels on the particle size of KTZ-NLCs; Figure S2: Response surface plots depicting the effects of (A) solid lipid and liquid lipid concentrations and (B) Span^®^ 80 and Tween^®^ 80 levels on the zeta potential of KTZ-NLCs; Figure S3: Cubes represent the suggested solution by DesignExpert^®^ software based on the criteria applied to the variables and responses during the optimization process; Figure S4: Polydispersity index of Ketoconazole-loaded NLCs (mean ± SD, N = 3); Figure S5: Drug content for Ketoconazole-loaded NLCs (mean ± SD, N =3); Figure S6: Entrapment efficiency for Ketoconazole-loaded nanostructured lipid carriers (mean ± SD, N = 3); Figure S7: pH values for Ketoconazole-loaded NLCs (mean ± SD, N = 3); Figure S8: (A) Zeta potential, (B) pH, and (C) Viscosity of the optimized mucoadhesive KTZ-NLC formulation over 90-day storage at 4 and 25 °C (mean ± SD, N = 3).
