# Supplementary material for: Mucoadhesive Nanostructured Lipid Carriers of Ketoconazole for Enhanced Dermal Delivery and Antifungal Activity: Formulation Optimization and In Vivo Evaluation
Source: Pharmaceutics. 2026 Jun 19;18(6):753. doi: 10.3390/pharmaceutics18060753 (PMC13307156; doi:10.3390/pharmaceutics18060753)
Supplement: Supplementary file 1 [file pharmaceutics-18-00753-s001.zip › pharmaceutics-4339770-supplementary.pdf]

Ketoconazole-Loaded Mucoadhesive Lipid Nanocarriers for Better Treatment Outcomes: Optimization, In-vitro, Ex-vivo, and In-vivo Evaluations.

Mashan Almutairi[1],*, Ahmed Adel Ali Youssef[2], Yazed S. Alsowaida[3], Ahmed Alobaida[1], Samir A. Ross[4].

[1]Department of Pharmaceutics, College of Pharmacy, University of Ha'il, Ha'il 81442, Saudi Arabia.

[2]Department of Pharmaceutics & Pharmaceutical Technology, Faculty of Pharmacy, Kafrelsheikh University, Kafrelsheikh – 33516, Egypt.

[3]Department of Clinical Pharmacy, College of Pharmacy, University of Ha'il, Ha'il 81442, Saudi Arabia.

[4]National Center for Natural Products Research, School of Pharmacy, The University of Mississippi, University, MS 38677, USA.

***Corresponding Author**

Mashan Almutairi, BSc, MSc, Ph.D.

Assistant Professor and Department of Pharmaceutics Chair

College of Pharmacy, University of Ha'il, Ha'il 81442, Saudi Arabia.

Email: m.almutairi@uoh.edu.sa

Tel: +966563555063

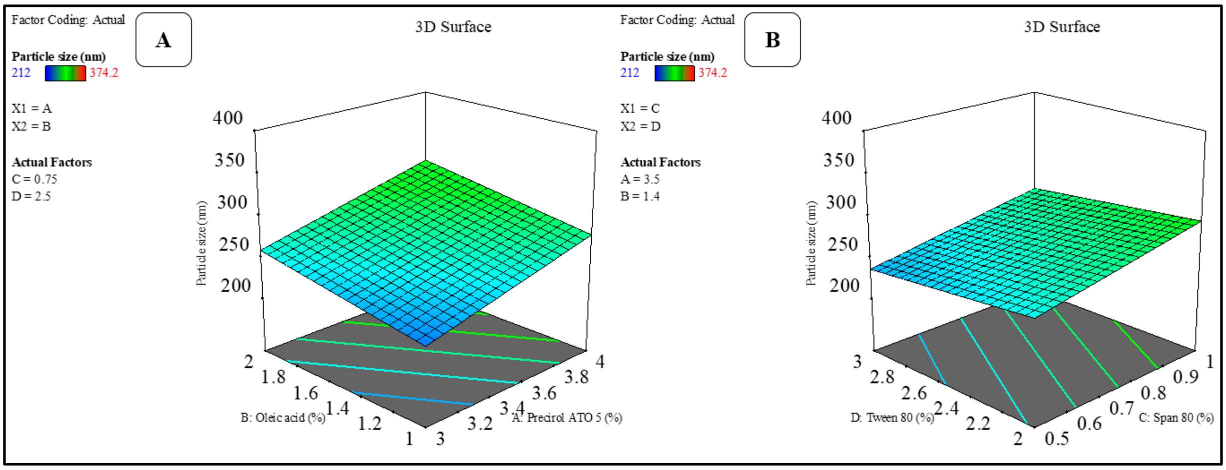

Figure S1. Response surface plots illustrating the effects of (A) solid lipid and liquid lipid concentrations, and (B) Span® 80 and Tween® 80 levels on the particle size of KTZ-NLCs.

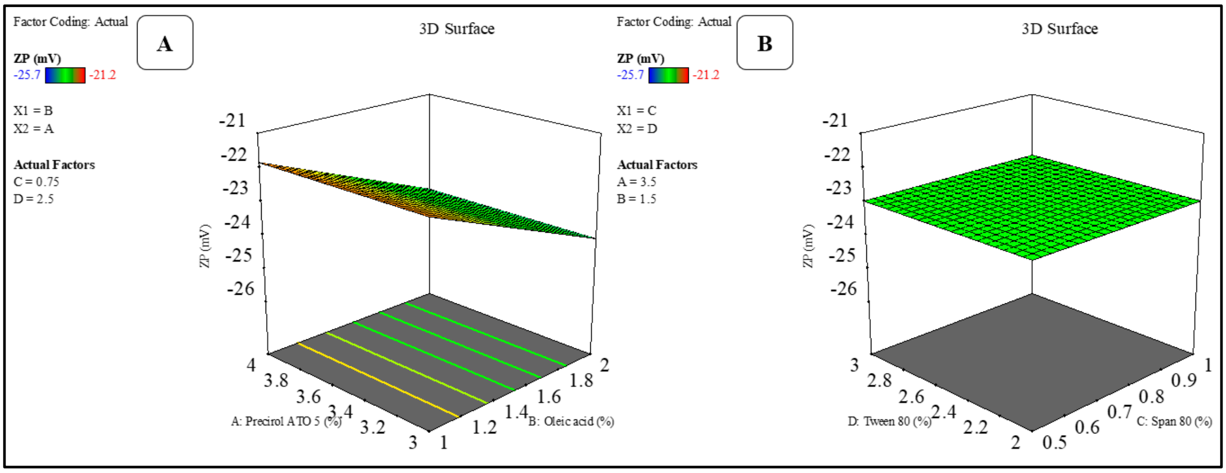

Figure S2. Response surface plots depicting the effects of (A) solid lipid and liquid lipid concentrations and (B) Span® 80 and Tween® 80 levels on the zeta potential of KTZ-NLCs.

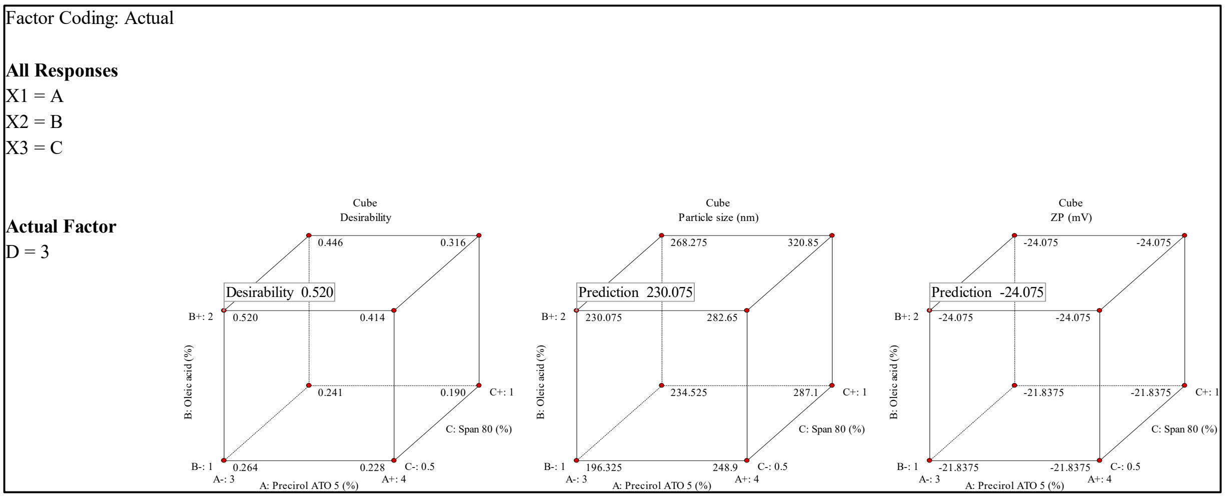

Figure S3 Cubes represent the suggested solution by DesignExpert® software based on the criteria applied to the variables and responses during the optimization process.

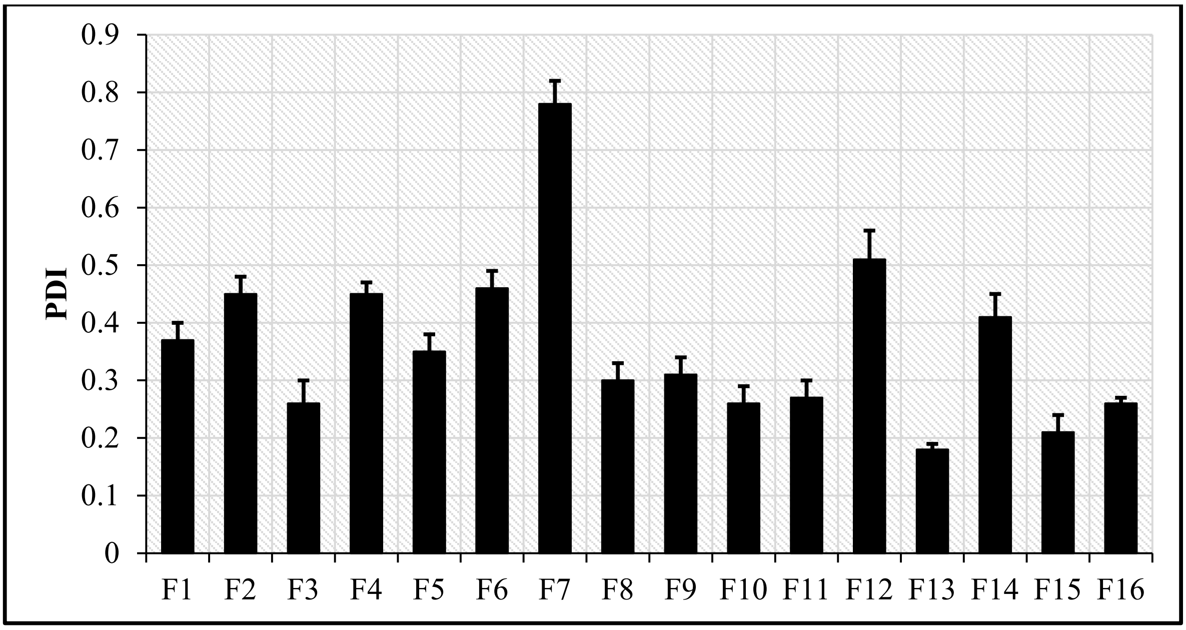

Figure S4 Polydispersity index of Ketoconazole-loaded NLCs (mean ± SD, n =3).

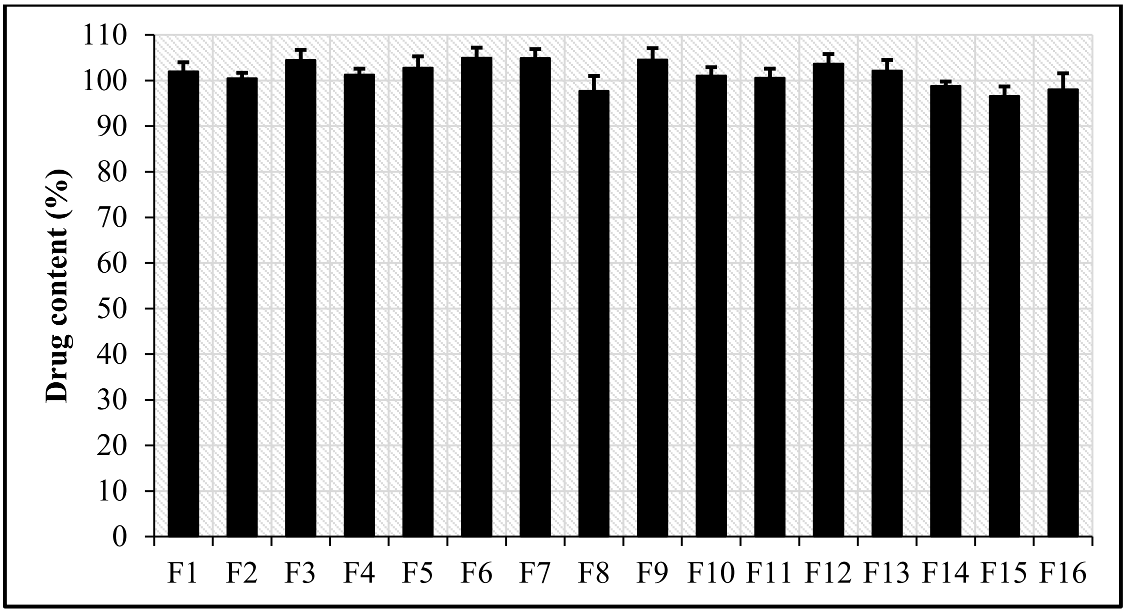

Figure S5 Drug content for Ketoconazole-loaded NLCs (mean ± SD, n =3).

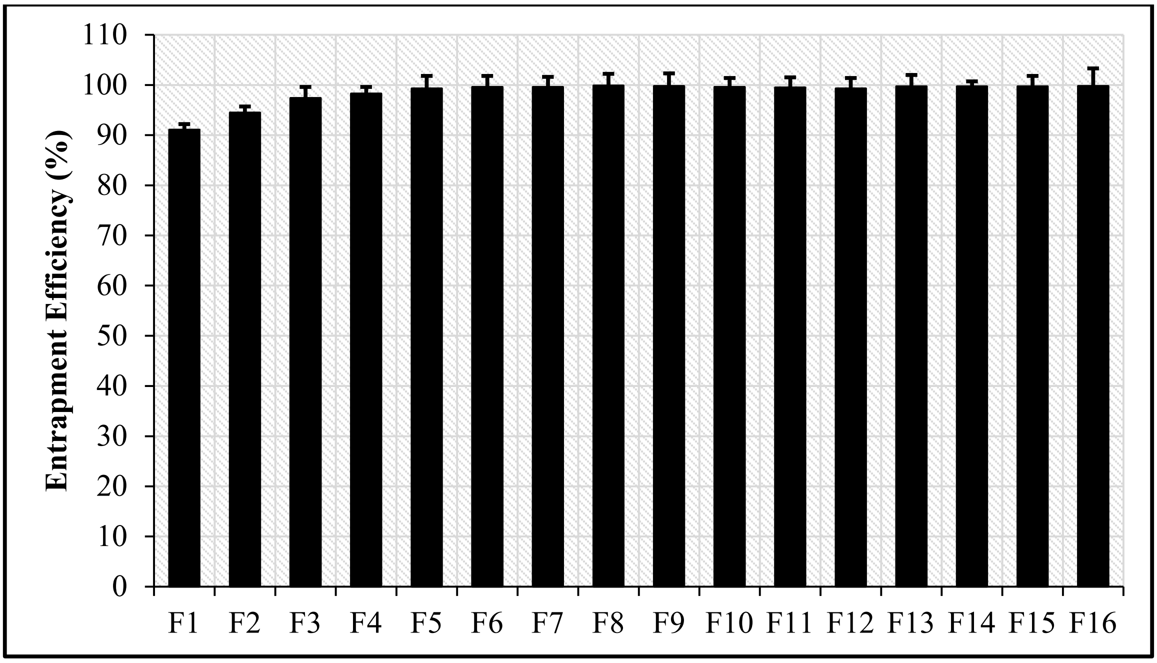

Figure S6 Entrapment efficiency for Ketoconazole loaded nanostructured lipid carriers (mean ± SD, n = 3).

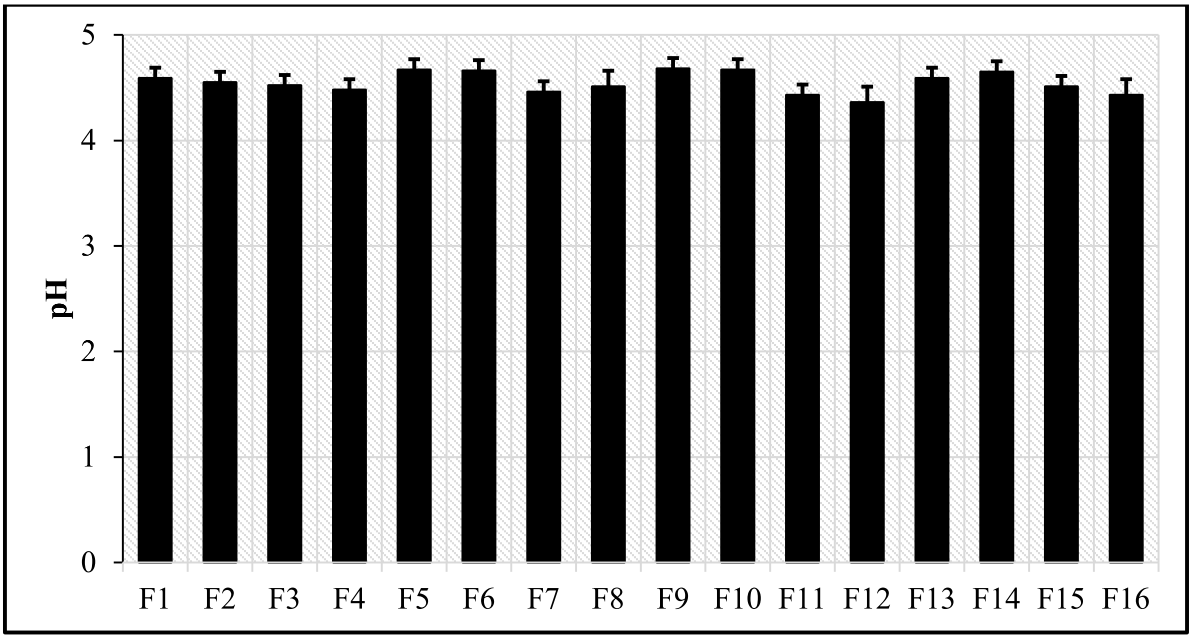

Figure S7 pH values for Ketoconazole-loaded NLCs (mean ± SD, n = 3).

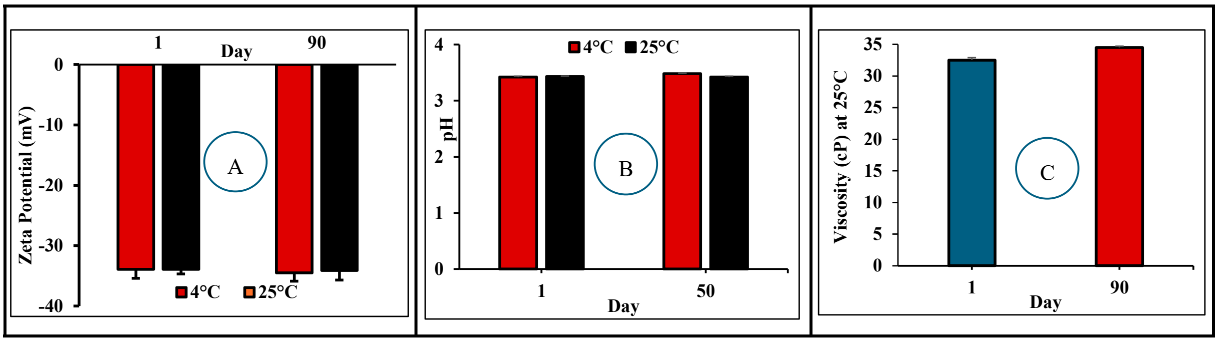

Figure S8 A) Zeta potential, B) pH, and C) Viscosity of the lead mucoadhesive KTZ-NLC-C formulation over 90-day storage at 4 and 25°C (mean ± SD, n=3).
